# Supplementary material for: Content-rich biological network constructed by mining PubMed abstracts
Source: BMC Bioinformatics. 2004 Oct 8;5:147. doi: 10.1186/1471-2105-5-147 (PMC528731; doi:10.1186/1471-2105-5-147)
Supplement: Additional File 5 — The original Chilibot query results of the term "long-term potentiation (LTP)" and 22 other terms, limiting the latest references analyzed to the years 1990, 1995, 2000, and 2004. [file 1471-2105-5-147-S5.bz2 › chilibotAdditionalFile5/ltp2000/html/PI-3K_NMDA.html]

 


 **PI-3K** and **NMDA** 
  
Found 7 abstracts in PubMed,  **7 abstracts were retrieved and analyzed**.  


---

 Search Google  |
 PDF files only 
|  EDU domain only 

---

**Interactive relationship** (e.g. stimulation, inhibition, etc)

- Inhibition of apoptosis by  **NMDA**  was blocked by the phosphatidylinositol 3 kinase PI 3 kinase  [ **PI-3K** ]  inhibitor LY294002, but it was unaffected by the mitogen activated protein kinase kinase inhibitor PD 98059.  Ref: 9756898 J Biol Chem, 1998
- These data demonstrate that the neurotrophic action of  **NMDA**  and its inhibition by ethanol are mediated by alterations in the activity of a PI 3 kinase  [ **PI-3K** ]  dependent antiapoptotic signaling pathway.  Ref: 9756898 J Biol Chem, 1998
- PI3 kinase  [ **PI-3K** ]  activity, which is stimulated by growth factors, is closely related to protective effects against NO and  **NMDA**  mediated toxicity in cortical neurons, especially those isolated from SHRSP.  Ref: 9166279 Lab Invest, 1997
- Inhibition of neuronal survival by ethanol corresponded with a marked reduction in the capacity of  **NMDA**  to increase the concentration of intracellular calcium, phosphorylate IRS 1, and activate PI 3 kinase  [ **PI-3K** ] .  Ref: 9756898 J Biol Chem, 1998
- The antiapoptotic action of  **NMDA**  was associated with an increase in the tyrosine phosphorylation of insulin receptor substrate 1 IRS 1, an increase in the binding of the regulatory subunit of PI 3 kinase  [ **PI-3K** ]  to IRS 1, and a stimulation of PI 3 kinase  [ **PI-3K** ]  activity.  Ref: 9756898 J Biol Chem, 1998

- :-)

  **Parallel relationship** (e.g. studied together, co-existance, homology, etc.)

  - BDNF increased phosphorylation of Akt, a target of PI 3 kinase  [ **PI-3K** ] , and  **NMDA**  also induced Akt phosphorylation, but only after an exposure that was long enough to induce BDNF expression.  Ref: 10212287 J Neurosci, 1999
  - In the absence of extracellular calcium,  **NMDA**  was unable to prevent apoptosis or to phosphorylate IRS 1 and activate PI 3 kinase  [ **PI-3K** ] .  Ref: 9756898 J Biol Chem, 1998
  - Neurons were subjected to several apoptotic paradigms, including serum deprivation, serum deprivation combined with exposure to  **NMDA**  receptor antagonists, or treatment with PI 3 kinase  [ **PI-3K** ]  inhibitors.  Ref: 10729337 J Neurosci, 2000
